# General and erosive tooth wear of 16-year-old adolescents in Kuantan, Malaysia: prevalence and association with dental caries

**DOI:** 10.1186/s12903-017-0451-9

**Published:** 2018-01-12

**Authors:** Noorhazayti Ab Halim, Rashidah Esa, Hooi Pin Chew

**Affiliations:** 1 Dental Public Health, Kulliyyah of Dentistry, International Islamic University Malaysia, Kuantan Campus, 25200 Kuantan, Pahang Malaysia; 2 Department of Community Oral Health & Clinical Prevention, Faculty of Dentistry, University of Malaya, 50603 Kuala Lumpur, Malaysia; 3 Department of Restorative Dentistry, Faculty of Dentistry, University of Malaya, 50603 Kuala Lumpur, Malaysia

**Keywords:** BEWE, Erosive tooth wear, Dental caries experience

## Abstract

**Background:**

The objective of this study was to determine the prevalence and severity of general tooth wear (GTW), i.e. tooth wear irrespective of etiology and erosive tooth wear (ETW), i.e. tooth wear predominantly due to erosion; and also to investigate the relationship between ETW and dental caries experience in 16-year-old adolescents in Kuantan, Malaysia.

**Methods:**

A multi-staged cluster sampling method was employed. A total of 598 16-year-old adolescents participated in this study. Participants’ demographic profile was assessed through a self-administered questionnaire. Clinical examinations were carried out under standardized conditions by a single examiner. The level of GTW was recorded using the modified Smith and Knight’s Tooth Wear Index (TWI) whilst ETW were recorded using the Basic Erosive Wear Examination (BEWE) index. This index was developed to record clinical findings and assist in the decision-making process for the management of erosive tooth wear. Dental caries was recorded using the D_3_MFT index whereby D_3_ denotes obvious dental decay into dentine detected visually.

**Results:**

The prevalence of GTW, ETW and dental caries, i.e. percentage of individuals found to have at least one lesion, was 99.8%, 45.0% and 27.8% respectively. Two thirds of affected teeth with GTW were observed to have a TWI score of 1 whereas almost all of the affected teeth with ETW had a BEWE score of 2. The mean D_3_MFT was 0.62 (95% CI 0.50, 0.73) with Decayed (D) teeth being the largest component, mean D_3_T was 0.36 (95% CI 0.30, 0.43). There was no significant association between socio-demographic factors and prevalence of ETW. Logistic regression analysis also showed no significant relationship between the prevalence of ETW and D_3_MFT (*p* > 0.05).

**Conclusions:**

Almost all adolescents examined had GTW but they were mainly early lesions. However, nearly half were found to have ETW of moderate severity (BEWE score 2). No significant relationship between the occurrence of erosive tooth wear and caries was observed in this population.

## Background

Tooth wear is a relatively new emerging dental public health problem which has not yet raised sufficient awareness among the public. Even dental professionals are not giving sufficient attention to this issue. Most dental professionals overlook the early stages of tooth erosion and dismissed tooth surface loss as something that is ‘normal’ or physiological and thus does not require any intervention [1]. The terms ‘tooth wear’ and ‘dental erosion’ had been used interchangeably by some whilst others used tooth wear as the cumulative effect of abrasion, attrition and dental erosion [2]. In addition, the terms dental erosion and dental erosive wear had often been considered to be synonymous. Huysmans et al. attempted to differentiate the two by defining, erosion as a partial demineralization of enamel or dentine by intrinsic or extrinsic acids and erosive tooth wear as the combined effect of erosion and mechanical wear (abrasion or attrition) on tooth surface [3] with erosion being the dominant process.

Epidemiological studies on the prevalence of tooth wear and erosive tooth wear had been conducted all over the world but the findings are not easily comparable due to the wide range of indices used [4, 5]. The most frequently used index was the Smith and Knight’s Tooth Wear Index (TWI) which was designed to record and assess tooth wear indiscriminate of aetiology [6]. Lussi’s index [7] and the Basic Erosive Wear Examination (BEWE) index [8] on the other hand had also been widely used in observational studies [9–11] frequently in the recording of wear lesions that are of erosive origin.

Recent studies of prevalence of tooth wear in pre-school children between the age of 3 to 6, have shown to range from 13.3% [12] to 51.6% [13], and 15.7% [9] to 75% [14] in 12 year old primary school children. The prevalence of erosive tooth wear in adolescents ranges from 14% in Denmark [15], 30.7% in Iceland to 100% in the United Kingdom [16]. Considering the short length of time the permanent teeth has erupted in children and adolescents and the rate of tooth wear that has been reported, it has been regarded as a potential public health issue.

In Malaysia, the first tooth wear study was conducted in 1996 by Milosevic and Lo [17] in Sabah, Borneo with 148 participants from a wide range of age (14–77-years). Ten years later, Saerah et al. [18] carried out an epidemiological study on tooth wear among 688 sixteen-years old schoolchildren in Kelantan, Peninsular Malaysia. Milosevic and Lo reported a prevalence of 95% and 41% of moderate and severe tooth wear respectively while Saerah et al. [18] reported a prevalence of 100% tooth wear. These studies were conducted at least 10 years ago on subjects with different age, sociodemographic and geographical backgrounds in Malaysia and both of them used TWI to record tooth wear indiscriminate of its etiology. There is a lack of local prevalence data on erosive tooth wear, which has a predominant erosion component [3]. Therefore, it is timely to investigate the prevalence of erosive tooth wear in Malaysia, and also to investigate this condition along with tooth wear indiscriminate of its etiology. Thus far, no studies have been conducted locally or elsewhere to investigate both conditions in the same cohort. Investigating these two conditions together may shed light on the differences of prevalence and severity of these two conditions and also facilitate comparison with other previously reported tooth wear studies conducted using different indices. We will henceforth refer to tooth wear with an erosive component as erosive tooth wear (ETW) [3] and tooth wear indiscriminate of aetiology as general tooth wear (GTW).

In addition to investigating GTW and ETW simultaneously, it is also useful to determine the prevalence of dental caries in the same cohort and whether there is an association with tooth wear. Two recent studies on Scandinavian adult population [19, 20] had shown that presence of erosive wear was positively associated with caries experience. Alaraudanjoki et al. [19] postulated that this could be due to common etiological factors between erosive wear and dental caries such as high or constant intake of sweetened soft drinks and low salivary secretion. However, other studies on children had found no such associations [21, 22].

Thus, the main objectives of this study were to determine the prevalence and severity of GTW and ETW among 16-year-old adolescents in Kuantan, Malaysia. Additionally, the secondary objective of this study was to relate prevalence and severity of ETW and GTW to the caries experience in the same adolescents.

## Methods

### Study population

This cross sectional study was conducted in Kuantan, Pahang, which is the most populous district in the east coast of Peninsular Malaysia. Kuantan was chosen as it is situated on the east coast of peninsular Malaysia i.e. geographically close to Kota Bahru and its population has similar socio-demographic profile as Saerah et al.’s [18] cohort. The conduct of the study follows the STROBE guidelines [23]. Ethics approval was obtained from the Medical Ethics Committee of the Faculty of Dentistry, University Malaya prior to the commencement of the study (DF CO1113/0071(P). Permission to conduct the study was also obtained from the Ministry of Education and the State Education Department.

### Sampling procedure

Convenience sampling was undertaken and Kuala Kuantan 1, which is the largest sub district, was selected out of the seven sub districts in Kuantan. A multistage cluster sampling was performed to select the schools. Out of 37 secondary schools, 15 schools met the inclusion criteria of which 5 schools with high enrolment of 16-year-old students were selected from the cluster. The sample size was calculated using a single proportion formula [24] at a precision of 5% with 95% CI and the expected prevalence of 20% from a previous tooth wear study by Saerah et al. [18]. In consideration of the precision needed, another 20% was added to increase the response rate, and a further design effect of two was set to increase the variance of estimate in cluster sampling [25, 26]. The sample size required was found to be 590. Prior to the oral examination, the consent form and Patient Information Sheet were sent to the parents of students between 15 and 16 years old, requesting permission for their children’s participation. Consent from students with written parental consent were also attained before they were included in the study. Students found to have oro-facial anomalies, undergoing orthodontic treatment and with poor oral hygiene were excluded from the study.

### Clinical examination

The oral examinations were carried out by a single calibrated examiner (NAH) in the schools under standardized conditions and universal cross infection preventive measure was adopted. The students were instructed to brush their teeth before being examined. The oral examinations were conducted at the school, using a portable dental chair and under the illumination of surgical head lights. Disposable mouth mirrors and ball-ended probes were used and the teeth were dried using gauze before examination.

Training and calibration exercises with a restorative specialist (CHP) on both the Tooth Wear Index (TWI) and Basic Erosive Wear Examination (BEWE) were undertaken before commencement of the study. Initial training involved clinical photographs of teeth with GTW and ETW of varying degrees of severity. Discussions were made between the researcher and the restorative specialist to resolve any ambiguities. Calibration was then performed on a different set of clinical photographs. The inter-examiner Cohen’s kappa values for the TWI and BEWE were 0.82 and 0.80 respectively, indicating very good agreement [27]. A second calibration exercise was conducted on twelve students who were randomly selected in one of the 5 schools. The same restorative specialist and the researcher examined the 12 students under the study conditions mentioned above. The inter-examiner Cohen’s kappa values was 0.87 indicating an almost perfect agreement [27]. In addition, intra-examiner reliability was also checked through duplicate examination of every fifth student until a total of 37 students (7% from the total sample size) [28], was attained. Kappa value for the intra-examiner reliability was 0.90 indicating an almost perfect agreement [27].

### Indices and diagnostic criteria used

General tooth wear (GTW) was recorded based on the Smith and Knights’ Tooth Wear Index (TWI) [6] (Table 1), erosive tooth wear (ETW) with the Basic Erosive Wear Examination (BEWE) index [8] (Table 1) and caries with the DMFT index based on the Oral Health Surveys: Basic Methods by WHO [28]. The D component at the threshold of D_3_ [29] denotes obvious dental decay into dentine detected visually (Table 1) and non-cavitated early caries lesions were not considered. No radiographs were taken. All forms of tooth wear i.e. non-carious tooth surface loss were included into the recording of GTW as the TWI was designed to be an all-inclusive wear index. However, only lesions that fits the diagnostic criteria previously published to be suggestive of erosive wear [7] were included in the recording of ETW. The criteria include early lesions with loss of tooth shine, cervical concavity with its width exceeding depth, cupping on the occlusal surface with rounded cusps among others.

**Table 1:** DMFT Index, Smith and Knight’s Tooth Wear Index and Basic Erosive Wear Examination Index

DMFT INDEX		SMITH & KNIGHT TWI		BEWE INDEX	
CODE	CONDITION	GRADE	CONDITION	GRADE	CONDITION
0	Sound	0	No loss of enamel surface on B/L/O/I, no change in contour on Cervical	0	No erosive Tooth Wear
1	Decayed	1	Loss of enamel on B/L/O/I, minimal loss of contour on Cervical	1	Initial loss of surface texture (enamel)
2	Filled, with decay				
3	Filled, without decay				
4	Missing due to caries	2	Loss of enamel exposing dentine <1/3 of the B/L/O/I, Defect less 1 mm deep on Cervical	2	Distinct defect, hard tissue <50% of the surface area (dentine)
5	Missing, any other reason				
6	Fissure Sealant	3	Loss of enamel exposing dentine >1/3 of B/L/O/I, Defect 1–2 mm deep on Cervical	3	Hard tissue loss > 50% of the surface area
7	Bridge abutment, crown or veneer/implant				
8	Unerupted tooth	4	Complete loss of enamel or pulp exposure on B/L/O/I, defect >2 mm deep Cervical		
T	Trauma				

All permanent teeth present were examined but teeth with large restorations involving multiple surfaces were excluded. Smith and Knight [6] proposed that the TWI index records is used to record four surfaces per tooth, which were the buccal/ labial, lingual/palatal, occlusal/incisal and cervical surfaces. However in this study, a modified version of the TWI index was used whereby only three surfaces per tooth were recorded. The surfaces were the buccal/labial, palatal and occlusal/incisal surfaces and the cervical areas were included as a part of the buccal surfaces. For the BEWE index, the highest score observed from each sextant was recorded [8]. The lower lingual surfaces were excluded in this study due to saliva pooling and inadequate lighting.

### Statistical analysis

Data was analysed using the SPSS version 19.0. Descriptive statistics such as frequency distribution and cross tabulation was conducted to determine the prevalence and severity of GTW and ETW. Logistic regression analysis was performed to test the association of ETW with socio-demographic characteristics such as gender, ethnicity, education level of parents and total household income of the family. The relationship between ETW with dental caries was also assessed with logistic regression analysis. The agreement between inter-examiner and intra-examiner for the TWI and BEWE scores, were analysed using Kappa statistic.

## Results

Overall 598 out of 790 schoolchildren were eligible for this study providing a response rate of 75.7% with 228 boys and 370 girls. Only adolescents with written consent were included in the study. More than half of the participants were Malays (329 or 55%), followed by Chinese (245 or 41%) and Indians (2 or 4%). Almost two thirds (61%) of the parents’ highest education level was secondary education. More than half (53.7%) of participants were from low to middle income families with a total household income of less than MYR2999.

### Prevalence and severity of GTW and ETW

The prevalence of GTW, i.e. percentage of individuals found to have at least one lesion, was 99.8% (597) and only one out of the 598 adolescents examined did not present with any tooth wear. The prevalence of ETW was 45.0% (269).

A total of 16,521 teeth were examined in this study and at the tooth level, almost two thirds of these teeth were observed to have a TWI score of 1 (Table 2). The distribution of affected tooth surfaces with GTW is shown in Table 2. The most frequently affected surface is either the incisal or occlusal surface whilst none of the palatal surfaces were affected. None of the teeth had GTW on more than two surfaces.

**Table 2:**
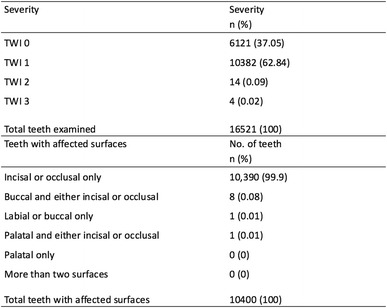
The severity of GTW at the tooth level and the distribution of affected tooth surfaces

**Fig. 1:**
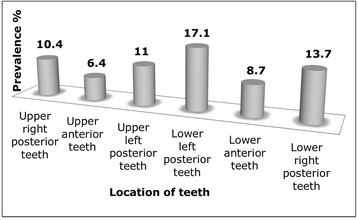
Prevalence of ETW according to sextants

The prevalence of ETW at the tooth level was only 2.73% with almost all (97.27%) of the affected teeth showing a BEWE score of 2. Prevalence of ETW according to sextant is shown in Fig. 1. The two most affected sextants were sextants 4 and 6 (lower left and right posterior teeth). The least affected sextants were sextants 2 and 5 (upper and lower anterior teeth).

As shown in the figure, it was found that both the lower right and left posterior teeth are most affected with erosive wear whilst the upper and lower anterior teeth the least affected.

### Association between ETW with socio-demographic characteristics

No significant association between socio-demographic factors and prevalence of erosive tooth wear was observed (Table 3). However, of all factors investigated, the level of parents’ education showed potential association whereby those with parents’ with tertiary education were 2.6 times more likely to have more than one erosive lesion.

**Table 3:** Association between ETW with socio-demographic characteristics

Variables	Adjusted Odds Ratio (OR) (95% CI)	*p*- value
Gender		
Male^a^		
Female	0.782 (0.556–1.101)	0.159
Ethnic group		
Malay^a^		0.947
Chinese	1.105 (0.765–1.596)	0.595
Indian/Punjabi/Pakistani	0.922 (0.383–2.220)	0.856
Parents’ highest education level		
No formal education^a^		0.307
Primary education	1.493 (0.248–8.980)	0.661
Secondary education	1.772 (0.329–9.530)	0.505
Tertiary education	2.558 (0.458–14.294)	0.285
Total household income (month)		
RM999 and below^a^		0.549
RM1000- RM2999	1.373 (0.831–2.268)	0.216
RM3000- RM4999	1.100 (0.633–1.912)	0.735
RM5000 and above	1.325 (0.712–2.465)	0.375

^a^Reference

Significant level set at *p* < 0.05

### Prevalence and severity of dental caries and association with erosive wear

The caries prevalence was 27.8%. The mean D_3_MFT was 0.62 (95% CI 0.50, 0.73); D_3_T = 0.36 (95% CI 0.30, 0.43), MT = 0.05 (95% CI 0.03, 0.07), FT = 0.21 (95% CI 0.15, 0.28) with Decayed (D_3_) teeth being the largest component. It was found that 27.5% with ETW had evidence of dental caries. However, logistic regression analysis showed no significant relationship between the prevalence of ETW and D_3_MFT (*p* > 0.05) (Table 4).

**Table 4:** Association between ETW with D_3_MFT

Variables	Adjusted Odds Ratio (OR) (95% CI)	*p*-value
Erosive Tooth Wear		
BEWE score 0^a^		
BEWE score > 0	0.973 (0.679–1.395)	0.884

^a^Reference

Significant level set at *p* < 0.05

## Discussion

Terms such as tooth wear, dental erosion and erosive wear had been used interchangeably in many previous studies, and therefore care should be taken regarding their interpretation. In this study, attempts were made to distinguish wear lesions that has an erosive component from those with exclusive mechanical etiologies such as attrition or abrasion by adopting the diagnostic criteria developed by Lussi [7]. Smooth bordered wear was ascribed to tooth erosion, while sharp-bordered faceted occlusal wear with matching wear in the opposing tooth was regarded as pure attrition and sharp-bordered, wedge shaped lesion on the cervical region was regarded as pure abrasion.

It is noteworthy that a huge discrepancy was observed between the prevalence of GTW and ETW. Although almost all subjects were found to have at least one GTW lesion, it is of low severity (TWI Score 1). This low severity coupled with its distribution mainly found on the incisal edges of lower incisors may indicate that it is only the manifestation of physiological wear. ETW however was observed in only almost half of the examined subjects but with higher severity (BEWE Score 2). This discrepancy highlights once again that comparing existing published prevalence data that were reported based on different indices is unjustified and efforts to establish relationship between potential dietary risk factors and general tooth wear (i.e. tooth wear indiscriminate of etiologies) had produced contradictory results.

Sixteen-year-old adolescents were selected because it is comparable to the previous local study that had been conducted by Saerah et al. [18] in Kelantan, Malaysia. Moreover, adolescents are recognized as having high aesthetic desire and awareness, potentially high caries rate, tendency for poor nutritional habits and eating disorders as well as unique social and psychological needs [30]. Another reason was because tooth wear is a new emerging problem locally among adolescents due to a changing lifestyle, including improved oral hygiene attitude, a growing consumption of healthy but erosive food and juices, and also an accessibility to acidic drinks.

Saerah et al. [18] in their study of 16-year-old schoolchildren classified tooth wear into raw and pathological tooth wear. They had defined pathological wear as wear that is not due to ageing and raw tooth wear as any detected wear lesions irrespective of etiology. Thus, their raw tooth wear is comparable to GTW of this study. In this study, the prevalence of GTW was 99.8% and concurs with Saerah et al’s report of 100% prevalence of raw tooth wear. However, the prevalence of pathological tooth wear reported by Saerah et al. was 20.1%. This could not be readily compared with ETW of this study. This is because although the investigators had referred to pathological tooth wear as the amount of wear that accelerated physiological tooth wear, it is unclear what was the criteria used to deem a lesion as being accelerated. Another local study [17] had reported prevalence of 95% and 41% of moderate and severe tooth wear respectively but comparison was also difficult as the cut-offs between these degrees of severity were not clear. In addition to that, their samples were from a wide age group and from a distinctly different geographical background and hence had very different dietary habits and lifestyles.

To date, there is no available literature on erosive tooth wear in Malaysia and the present study can be regarded as the first erosive tooth wear study in Malaysian adolescents. The prevalence of erosive tooth wear (45%) in this study was found to be higher than two comparable studies conducted on 15–16 year adolescents in Iceland and The Netherlands [9, 31]. In both of these studies, all teeth were included in the examination and Lussi’s index [7], which is designed specifically for recording of erosive wear, was used and both of these studies reported a lower prevalence of 30.7% and 30% respectively. This could be because Score 1 of both the indices are challenging to detect, especially in a compromised examination environment, and the lesions detected were predominantly score 2 lesions. The threshold for Score 2 of BEWE and Lussi’s index are also different. The former uses breadth of affected area regardless of whether dentine was exposed or not as a criterion while the latter utilizes the presence of dentine exposure as the main criteria. It is to be expected that the occurrence of dentine exposure (Lussi‘s index) happens at a later point. However, the discrepancy of their findings from ours may also be a reflection of the actual geographical and population differences.

There is a lack of regional study that was conducted on 15–16 year olds and the most comparable one to this study would be the one by Wang et al. on 12-year olds in Guangzhou, China [32]. They had used O’Sullivan’s index [33] and the diagnostic criteria described by Eccles [34] to record erosive wear. They reported low prevalence of erosion (27.3%). The lower prevalence recorded could be due to the younger age group with less exposure to dietary risk factors, compared to the older age group in our study. Another regional study by Zhang et al. on 12-year olds in Hong Kong [14] however reported that 75% of the children had some sign of erosion. Although they had used BEWE to record the erosion status of the children, the authors had emphasized that the wear recorded in their study did not necessarily have a dental erosion component, as no specific diagnostic criteria for erosive wear were used. Therefore, Zhang et al’s report of 75% prevalence is more comparable to our general tooth wear findings rather than erosive wear.

In a recent observational, cross-sectional study across seven European countries, Bartlett et al. [35] found that 57.1% of young adults had tooth wear that is irrespective of aetiology, which is by definition similar to GTW of this study, with the BEWE index. This is lower than our 99.8%, even though our cohort is younger. This is most likely because of the different severity threshold criteria between BEWE and TWI and hence such comparison is unjustified. However, it is interesting to note that when compared to the prevalence of ETW of this study, it is higher than our cohort, despite only scoring the oral and facial surfaces and not using any diagnostic criteria.

We observed that the lower posterior teeth were most frequently affected by ETW (Fig. 1) and this observation concurred with other studies that specifically investigated the prevalence of erosive wear [9, 31]. Arnadottir et al. [9] reported that the most common clinical manifestation of erosion was the appearance of cup-like lesions on the cusps of lower first molars whilst van Rijkom et al. [31] found that first molars and upper anterior teeth were affected predominantly. The higher distribution of erosive wear on molars had been attributed to the chronology of eruption and hence the cumulative time these teeth had been exposed the oral environment [9]. However it is still unclear why lower molars were more affected by erosive wear than the upper molars and requires further investigations.

Previous studies had observed that there could be an inverse relationship between the occurrence of dental erosion and dental caries. Honorio et al. [36] found that in an environment where there were concurrent erosive and cariogenic challenges, demineralization were less compared to when there was erosive or cariogenic challenges alone. It was hypothesized that in a highly acidic environment (< pH 4.4) rendered either by intrinsic or extrinsic acid, bacterial metabolism is altered [36] and even suffer from acidic death [37]. In this study, we did not find any significant relationship between the prevalence of ETW and caries. However, caution has to be taken in interpreting this result as the non-significance could partly be contributed by the low prevalence and severity of caries in the cohort of this study and the protocol of this study. The caries prevalence and severity (27.8%, D_3_MFT = 0.62) in this study population were much lower than the national average (59.0%, DMFT = 2.28) and almost similar to the UK for prevalence but much lower than in other countries such as China, Australia, New Zealand, USA and Turkey [38]. In addition to that, the inclusion threshold of caries used was cavitated caries into dentine (D_3_MFT) and bite-wing radiographs were not included. Incipient caries lesions and approximal lesions were not included and therefore there could be under-estimation in the reported prevalence of caries.

The findings of this study were limited to adolescents in Kuantan and the results could not be generalized to adolescents in Malaysia. Both the cohort of this study and that of Saerah et al.’s [18] were from the east coast of peninsula Malaysia and have different socio-demographic profile from that of the west coast of the peninsula and the Borneo island. Hence future studies should be conducted in other populations in Malaysia. Additionally, another significant limitation of the study was the relatively longer time needed to complete the intra-oral examination for each participant, because three indices were used on all surfaces of all teeth that were present. This had caused discomfort to the participants and increased the rate of examiner’s fatigue. Since Saerah et al. and this study had revealed that GTW though prevalent, was not severe among adolescents, it may not be necessary to conduct prevalence study on GTW and only limit future studies on ETW.

## Conclusion

It can be concluded that general tooth wear among adolescents in Kuantan, Malaysia is highly prevalent but they were mainly early lesions. However nearly half of the adolescents exhibited erosive tooth wear of moderate severity. Further studies should to be done in populations in other geographical area and dietary risk factors that could be associated to the observed prevalence should be explored.

## Abbreviations

B: Buccal
BEWE: Basic Erosive Wear Examination
D: Decayed teeth
D_3_MFT: Decayed (Obviously cavitated), Missing, Filled Teeth
D_3_T: Decayed (caries into dentin) teeth
ETW: Erosive tooth wear
GTW: General tooth wear
I: Incisal
L: Labial
O: Occlusal
P: Palatal
TWI: Smith and Knight’s Tooth Wear Index
WHO: World Health Organization
